# Commentary: Circulatory pattern of cytokines, adipokines and bone markers in postmenopausal women with low BMD

**DOI:** 10.3389/fimmu.2019.02666

**Published:** 2019-11-14

**Authors:** Carmen Mannucci, Gioacchino Calapai, Sebastiano Gangemi

**Affiliations:** ^1^Department of Biomedical and Dental Sciences and Morphofunctional Imaging, University of Messina, Messina, Italy; ^2^School and Division of Allergy and Clinical Immunology, Department of Experimental Medicine, University of Messina, Messina, Italy

**Keywords:** osteoporosis, IL-31, IL-33, IL31/33 axis, post-menopausal osteoporosis, inflammation

Osteoporosis, one of the most important conditions associated with aging, is characterized by low bone mass and micro-architectural bone tissue deterioration, due to bone resorption and bone formation imbalance (1, 2). The immune system contributes to postmenopausal osteoporosis through the pro-inflammatory cytokine modulation of osteoblast and osteoclast activity (3–6). It has also been suggested that adipose tissue influences the regulation of bone metabolism, contributing to osteoporosis pathophysiology, thanks to the independent endocrine and paracrine activity associated with adipokines production (7, 8).

We have read with great interest the article by Azizieh et al. (8), aimed at measuring circulatory levels of several cytokines (IL-1β, IL-4, IL-6, IL-8, IL-10, IL-12, IL-13, IL-17, TNF-α, IFN-γ, TGF-β), adipokines, bone turnover marker density, and estradiol levels in postmenopausal women with normal and low bone mineral density (BMD). Results showed that circulatory cytokine levels were comparable in women with low or normal BMD. Women with low BMD, showed statistically significant higher median circulatory levels of adipokines compared to women with normal BMD. Moreover, while the C-terminal cross-linking telopeptide of type I collagen (CTX) levels were not different between the two groups, the procollagen type I N Propeptide (PINP), PINP/CTX ratio, and estradiol levels were significantly lower in women with low BMD. The Adiponectin, PINP, PINP/CTX ratio, and estradiol levels correlated significantly with BMD of the hip and spine (8).

We congratulate the authors for a remarkable study, and agree that the results indicate the possible role of the considered cytokines, adipokines, and bone turnover markers in the pathogenesis of postmenopausal osteoporosis. Here we focus on the role of the IL-31/IL-33 axis.

Interleukin-33 (IL-33) belongs to the IL-1 cytokine family, it is mainly expressed in stromal cells, and upregulated following pro-inflammatory stimulation (9).

IL-33, described as a Th2 cytokine inducer, is considered a traditional cytokine, an “alarmin” and a nuclear factor controlling gene transcription (10). IL-33 has powerful effects on many cell types such as ILC2s (type 2 innate lymphoid cells), mast cells, eosinophils, and Th2 lymphocytes, in particular, IL-33 plays a major role in ILC2 recruitment. IL-33 activates ILC2s via NF-κB and MAPK signaling pathways, causing enhancement of the production of cytokines, chemokines, and peptides (11–15). The responsiveness to these cytokines depends on resident tissues and species, and co-stimulatory cytokines are required for activation (16).

It has also been demonstrated that IL-33 increases its expression after cell death, resulting in the induction of other cytokines including IL-31 (10).

Interleukin-31 (IL-31), belongs to the gp130/IL-6 cytokine family expressed by activated memory CD45RO+ T lymphocytes skewed toward a Th2 phenotype (17–19). Scientific evidence shows a biological role for IL-31 in immunity and inflammation (17, 18). Several cytokines and transcription factors are involved in the development of osteoporosis, and some of them are regulated by IL-31 (18–21). Engagement of the receptor complex results in the activation of Janus kinase and different signaling molecules, including signal transducers and activators of transcription factors, Akt, NF-κB (12, 13, 18), MAPK (17, 18), and the PI3K signaling pathways (19). These pathways are involved both in bone remodeling and inflammation (20, 21).

In our previous studies, based on the IL-31 and IL-33 role in inflammation and bone remodeling (22–24) we evaluated, their involvement in postmenopausal osteoporosis. Measurements of the serum of IL-31 and IL-33 were performed both in women with osteoporosis and healthy women as controls. Osteoporosis was evaluated by BMD measurement expressed as T-score (23).

Our results showed a statistically significant increase of IL-31 serum levels in postmenopausal women with decreased BMD, suggesting a role of IL-31 in osteoporosis. Serum IL-31 levels are not related with the severity of osteoporosis, as indicated by BMD values and/or the presence of fractures (23).

Serum IL-33 levels were significantly lower in postmenopausal osteoporotic patients than non-osteoporotic patients and a correlation between IL-33 and PTH serum levels was also observed (24).

Though the direct effect of IL-33 on osteoclast function or bone resorption is not clear, our results showed that a negative correlation between IL-33 and CTX seems to confirm the inhibition of osteoclast differentiation mediated by IL-33 (24).

The lower levels of IL-33 found in osteoporotic patients agree with several experimental observations showing inhibition of osteoclast differentiation by IL-33 (25–27). It is known that IL-33 inhibits RANKL-dependent osteoclast formation, protecting it from inflammatory bone loss (28, 29). Furthermore, IL-33 inhibits osteoclast differentiation by inducing antiosteoclastogenic cytokines such as IL-10, IL-4, and IFN-γ and granulocyte-macrophage colony-stimulating factor, skewing osteoclast precursors differentiation to alternatively activated macrophage and dendritic cells (25). A RANKL-like action of IL-33 has also been suggested in human osteoclast formation (30). IL-33 pro-resorption effects on bone is weak and highly variable, compared to the strong effect of RANKL, with some types of osteoclast progenitors that differentiate into functional resorbing cells consequent to IL-33 stimulation and other osteoclast progenitor-containing unresponsive populations (31).

IL-31 serum levels increase in aged osteoporotic patients, suggesting a link between bone resorption and cytokine overexpression, that could enhance bone resorption through chemokines and induction of proinflammatory osteoclastogenic cytokines, leading to osteoclast precursors recruitment, differentiation, and activation. Our results suggest a key role of IL-31 in senile osteoporosis but the exact mechanism of action remains unknown. A clear understanding of IL-31 involvement in bone resorption immunopathology can provide more effective strategies for the treatment of senile osteoporosis.

Our data suggest the existence of an IL-31 and IL-33 correlation axis. This is in accordance with other authors that showed an involvement of the IL-33/ST2 axis in the progression of several inflammatory diseases, influencing the generation of Th17, producing IL-31 (32, 33). The high serum level of IL-31 and IL-33 have also been reported in people with inflammatory and autoimmune diseases (33, 34). IL-31 and IL-33 are linked to each other; induction of one cytokine by the other is correlated with disease severity, inflammation, and harmful processes. Data suggest that IL-33/ST2 axis activation can be considered a biomarker of both a Th2/IL-31 and Th17 immune response (10, 34).

Our results can contribute to a better understanding of the mechanisms at the basis of the immune system capacity, that mount osteoclastogenic immune reactions through inflammatory responses, crucial in senile osteoporosis. Moreover, IL-33 could be considered an important bone-protecting cytokine, becoming a target in the prevention and therapy of postmenopausal osteoporosis.

## Author Contributions

CM, GC, and SG have contributed equally to the work, and approved the article for publication.

### Conflict of Interest

The authors declare that the research was conducted in the absence of any commercial or financial relationships that could be construed as a potential conflict of interest.
